# Conceptualization of Depression among Medical Students and Its Differences during Medical Education

**DOI:** 10.3390/epidemiologia5030042

**Published:** 2024-09-09

**Authors:** Santi Arana-Ballestar, Ricardo Campos-Ródenas, Beatriz Olaya, Javier Santabárbara

**Affiliations:** 1 Centro de Salud Parque Goya, 50018 Zaragoza, Aragón, Spain; 2 Hospital Clínico Universitario Lozano Blesa, 50009 Zaragoza, Aragón, Spain; 3 Research, Innovation and Teaching Unit, Parc Sanitari Sant Joan de Déu, 08950 Sant Boi de Llobregat, Barcelona, Spain; 4 Centro de Investigación Biomédica en Red de Salud Mental (CIBERSAM), 28029 Madrid, Comunidad de Madrid, Spain; 5 Faculty of Medicine, University of Zaragoza, 50009 Zaragoza, Aragón, Spain

**Keywords:** depression, psychological theories, concept formation, medical students, Mausdley Attitudes Questionnaire

## Abstract

The definition of mental disorders has been traditionally a matter of discussion, and it has relevant implications in research and healthcare. Our aim was to explore the conceptualization medical students have of depression and to determine differences across academic years. The Maudsley Attitudes Questionnaire was adapted through a double translation, double back-translation and a preliminary validation, obtaining a Spanish edition. All students of the Faculty of Medicine of the University of Zaragoza and doctors who graduated from this University in 2020 were invited to answer the online questionnaire, and we received 222 answers (response rate: 15.2%). The results were compared by years and levels of education using an ANOVA. The social realist, behavioral, biological, cognitive and psychodynamic models were the most endorsed. The psychodynamic and nihilist models were less embraced by students in later educational years. These students also reported greater confidence in their understanding of depression and of its biological, cognitive and behavioral models. In conclusion, the conceptualization of depression among medical students is complex and multidimensional, and appears to be similar across different years of education. In later years, we found less support for the psychodynamic model, increased confidence in psychiatry and greater ease in handling the concepts of its leading models.

## 1. Introduction

Mental disorders are a major contributor to the global burden of disease [1]. The greatest proportion of this burden corresponds to depressive disorders which, in Spain, affect about 5% of the general population. In this country, its incidence, disabling effects and the costs involved are increasing [1,2]. However, the adequate management of depression is hampered by the uncertainty surrounding its pathogenesis [1] and, more fundamentally, its conceptualization.

Indeed, the definition of “mental illness” has traditionally been a matter of discussion [3]. Human behavior includes a wide range of phenomena (from individual genetic predispositions to the influence of society and culture) whose integration and definition represents a major challenge [4]. This challenge is theoretical rather than empirical; it is not possible to distinguish the “real facts” from the concepts we use to understand them [5]. These concepts work as metaphors or heuristics that define the object of study and its properties. Therefore, the different definitions of mental illness include, tacitly, prepositions about its existence, nature, the possibilities of its study, problems to solve, etc. [5].

The shared commitment of a scientific community to a certain set of concepts, beliefs or heuristics is what the philosopher of science Thomas Kuhn defines as “paradigm” [6]. These assumptions create a philosophical framework which notably influences scientific practice. Several studies have shown that the concepts used to understand mental disorders can influence the recommendations for treatment [7] and the design and interpretation of their research [8]. As every philosophical framework has its own limitations, it is essential to know the perspective from which the clinical work or research is being developed [4].

The biomedical model represents the most dominant scientific paradigm in psychiatry [9,10]. It defines mental disorders as brain illnesses and, therefore, attempts to manage them through biological interventions (e.g., psychopharmacology). However, this model has been widely questioned from its beginning [9,11], with critical psychiatry one of the most questioning movements [12].

The validity and utility of the biomedical model is still under debate, with some of its criticisms arising from the lack of clinical innovation and poor mental health outcomes [10] and other theoretical and philosophical questions such as explanatory reductionism [13], the reification of diagnostic constructs [8], the dimensional vs. categorical approach [3] or the thresholds between health and illness [3]. The answer to many of the problems of psychiatry could be found through the analysis of the models, philosophies and concepts that underlie research and clinical practice.

For this reason, some authors consider “conceptual competence” as a moral responsibility of the discipline [4]. Despite its importance, its contents are not usually included in the education of medical students and residents [4,14]. Indeed, few studies have explored the concepts of mental health disorders used by mental health professionals [15]. Furthermore, if the perspective of mental health professionals can inform us about the current conceptual model of psychiatry, exploring the attitudes of medical students could give us clues about the future evolution of the discipline. Also, comparing the conceptualization of mental disorders of medical students from different academic years could help us determine the evolution of these concepts through their university degree.

Additionally, most prior research has employed different instruments that are mainly focused on comparing only two models: the biological model versus the psychosocial model. However, the “psychosocial model” is, in fact, a mixture of several different models.

One of the exceptions to these limitations is the work of Harland et al. [16], which was conducted on a sample of psychiatrists. In their study, they developed the Maudsley Attitudes Questionnaire (MAQ), which assesses the endorsement of eight different psychiatry models (biological, cognitive, behavioral, psychodynamic, social realist, social constructionist, nihilist and spiritual) applied to four mental disorders (schizophrenia, major depressive disorder, generalized anxiety disorder and antisocial personality disorder). This questionnaire has been applied to different populations (psychiatrists [16], psychologists [17] and laypeople [18]), therefore allowing their study and comparison.

The aim of our work is to begin to fill in the knowledge gap about medical students’ psychopathological conceptualization. In this study, we explore the concepts employed by Spanish medical students to understand depression and its evolution throughout their medicine degree by using the MAQ.

We decided to focus on depression for several reasons. As has been explained, depression is one of the most prevalent, disabling and costly mental disorders in the world and in our country. Therefore, it is one of the most studied disorders in medical school, and almost all physicians will encounter patients with depression. Finally, given the preliminary nature of this work, the use of the complete MAQ in this population would have been excessively challenging, and thus would have impacted on the rate and quality of their responses.

## 2. Materials and Methods

### 2.1. Study Design

This cross-sectional study was conducted at the University of Zaragoza through an online survey sent to a convenience sample of medical students between January and February 2021. The design of the study was revised and approved by the Research Ethics Committee of Aragón. Participation was anonymous and voluntary. The data were kept in password-protected computers and online file storage.

### 2.2. Participants

The questionnaire (delivered through the online platform Google Forms) was sent via email to all students of medicine of the University of Zaragoza and doctors who recently graduated, in 2020, from the same university (*N* = 1460, 71.6% female).

#### 2.2.1. Inclusion Criteria

Medicine students from the University of Zaragoza and medical doctors who graduated from this university in 2020.

#### 2.2.2. Exclusion Criteria

Students that reported having additional education or experience in mental health (e.g., psychology studies, other mental health studies, etc.).

#### 2.2.3. Clustering by Level of Education

In the University of Zaragoza, the study of psychiatry starts in the second semester of second year. Medical clerkship in different medical departments starts in the second semester of fifth year. Therefore, in order to control for possible variability across academic years, medical students were clustered into the following three groups: (1) first and second years were grouped in the pre-psychiatry level (level 1); (2) students from third to fifth year were included in the post-psychiatry level (level 2); and (3) sixth-year students and recently graduated doctors were included in the pre-residency level (level 3). Neither the academic program the subjects received nor the teaching staff underwent changes during the years of the study.

### 2.3. Instrument

The Maudsley Attitudes Questionnaire (MAQ) was originally developed by Harland et al. [16] to study how psychiatrists conceptualize mental disorders. It explores the endorsement of eight different models of psychiatry: biological, cognitive, behavioral, psychodynamic, social realist, social constructionist, nihilist and spiritual. Each model is represented by four items concerning the etiology, classification, treatment and research (see Table 1) of four disorders (as defined in the DSM-IV): schizophrenia, major depressive disorder (from now on, “depression”), generalized anxiety disorder and antisocial personality disorder. Attitudes are measured through a Likert scale, from 1 = “strongly disagree” to 5 = “strongly agree”.

The psychometric properties of this questionnaire, beyond the initial validation made by Harland et al. [16], are not fully known [17].

We focused the questionnaire on depression and excluded the other disorders for several reasons: the preliminary nature of this work, the basic education level of the students (in which depression is, probably, the best-known and most studied disorder) and for the sake of brevity, to achieve an acceptable rate of response.

### 2.4. Adaptation of the Instrument

Our method was designed based on the procedures recommended for the cross-cultural adaptation of self-report measures [19]. The process included a double and independent translation and back-translation, followed by a pilot study with ten medical students and a preliminary validation with two experts.

#### 2.4.1. Translation

Both the translation and the back-translation were made independently by a health professional and a professional translator.

#### 2.4.2. Pilot Study

The first version of the questionnaire obtained after the translation process was tested in a pilot study with ten sixth-year medical students. Based on previous studies [18], we included the item “I have a good understanding of depression” at the beginning of the questionnaire, and the sentence “I understand well this item” after each of the statements to evaluate their understanding of the instrument and the disorder. This addition was meant to assess the appropriateness of the questionnaire (designed by psychiatrists and intended for psychiatrists) for a less educated population, such as medical students. Their mean understanding of depression was 3.1 out of 5 and their understanding of the models ranged from 13.7 (cognitive) to 17.2 (social realist) out of 20.

We asked the students to complete the same task used by Harland et al. [16] to design and test the construct validity of the questionnaire. Participants were asked to assign each item to one of the eight models included in the questionnaire, obtaining a mean correct answer of 71.6% (59.4–84.4%). Considering that they were undergraduate students and that the questionnaire was originally designed for psychiatrists, this result was considered acceptable.

#### 2.4.3. Preliminary Validation with Experts

The same validation task was conducted by two experts, a psychologist and a psychiatrist, both of whom are university professors. The results yielded 90.6% correct answers, supporting the preliminary validity of the translated version.

#### 2.4.4. Final Design of the Spanish MAQ (MAQ-Esp)

As explained above, for the purpose of this study, only depression was included in the MAQ. The statements were adapted to match the subject “depression” instead of “disorder”. A section asking about sociodemographic data (age, sex, year of their degree) was added at the beginning, as well as a question about their additional experience of or knowledge about mental health.

### 2.5. Data Analysis

#### 2.5.1. Statistics

First, descriptive statistics were calculated. Then, we contrasted the normal distribution of the variables using the Kolmogorov–Smirnov test. Differences between educational levels were evaluated with the ANOVA test. To assess which results were statistically significant (*p* < 0.05), Bonferroni’s post hoc correction was used.

Statistical analyses were conducted with the IBM SPSS 26, Open Epi (https://www.openepi.com/Menu/OE_Menu.htm, accessed on 1 June 2021) and Excel 2013.

#### 2.5.2. Endorsement Measures

To more clearly illustrate the findings, and in line with previous studies [17,18], the figures we use represent endorsement scores that reflect the Likert rating scale of the questionnaire (1 = strongly disagree, 3 = neutral, 5 = strongly agree); these result from dividing the total scores by four, the number of item of each model. In the same vein, to clarify the interpretation of statistically significant results, Likert absolute differences (LADs) are reported, along with *p* values, on a scale from 1 to 5. Thus, for example, an LAD of +1 means the overall attitude towards the model has changed one point towards agreement (for example, from disagree to neutral, from neutral to agree, and so on).

## 3. Results

A total of 222 students answered the questionnaire (response rate: 15.2%). Six of them were excluded for not meeting the inclusion criteria, thus the final sample included 216 participants. This sample is described in Table 2. The pre-psychiatry level contained 57 students (89.5% female, mean age 19.2 years, SD = 0.9), 74 participants were included in the post-psychiatry level (75.7% female, mean age 22.2, SD = 1.8) and 85 in the pre-residency level (75.3% female, mean age 24.8, SD = 3).

### 3.1. Students’ Understanding of Depression and Models

Overall, the mean score of the item “I have a good understanding of depression” was above 3 out of 5. The scores were different for different levels (*p* < 0.001). Post hoc analyses showed that level 1 obtained lower scores than level 2 (LAD 0.67, *p* < 0.001) and level 3 (LAD 0.7, *p* < 0.001).

Taking into account all academic years, the scores for the understanding of models were above 13.5 out of 20. The scores of the biological, cognitive and behavioral models were significantly different across these levels (*p* < 0.001). Post hoc tests revealed that the reported understanding of biological and behavioral models was lower in level 1 than in level 2 (LAD 0.34, *p* = 0.007 and 0.54, *p* < 0.001) and level 3 (LAD 0.31, *p* = 0.012 and 0.51, *p* < 0.001). The score of the cognitive model was lower in level 1 than in level 2 (LAD 0.31, *p* = 0.046).

### 3.2. Models of Depression in Medical Students

**Figure 1:**
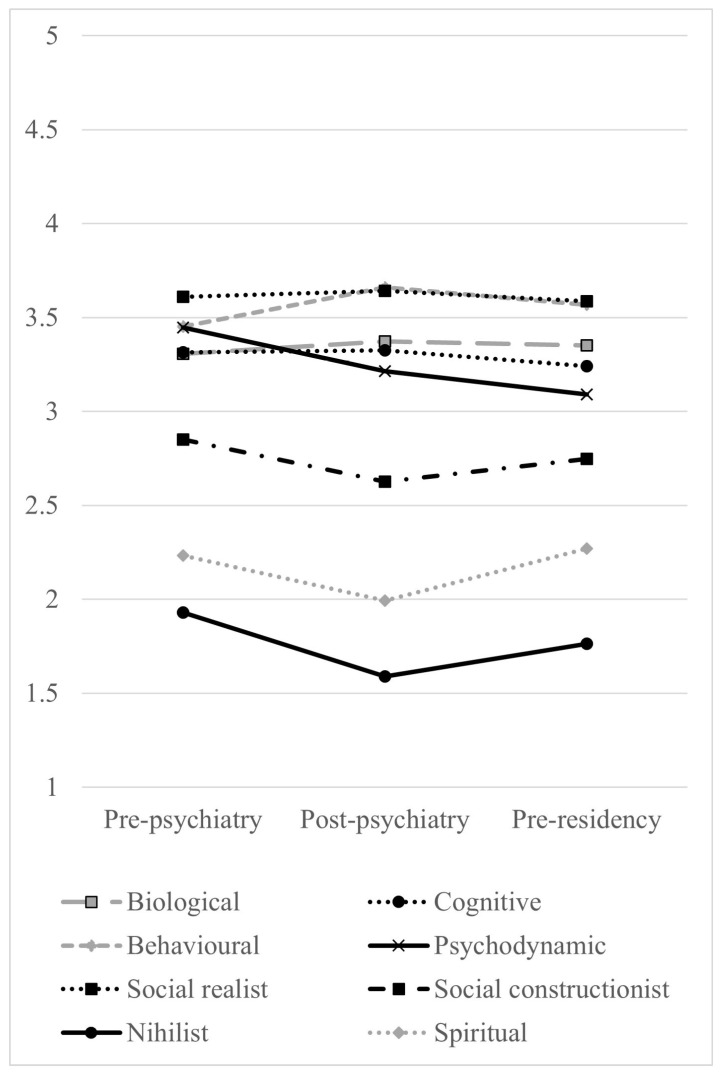
Mean aggregate endorsement scores by model and level of education. 1 = strongly disagree, 3 = neutral, 5 = strongly agree (see text).

Table 3 shows the mean scores of each model by level of education. As explained above, Figure 1 presents the mean endorsement scores to reflect the Likert rating scale of the questionnaire (1 = strongly disagree, 3 = neutral, 5 = strongly agree). The social realist and behavioral were the most endorsed models, followed by the biological, psychodynamic and cognitive models. Overall, the least endorsed models were, in descending order, the social constructionist, spiritual and nihilist models. A difference between levels was only found for the psychodynamic model. Post hoc tests revealed that the mean score of the psychodynamic model was higher in the pre-psychiatry level than in the post-psychiatry level (LAD −0.23, *p* = 0.039) and pre-residency level (LAD −0.36, *p* < 0.001).

**Figure 2:**
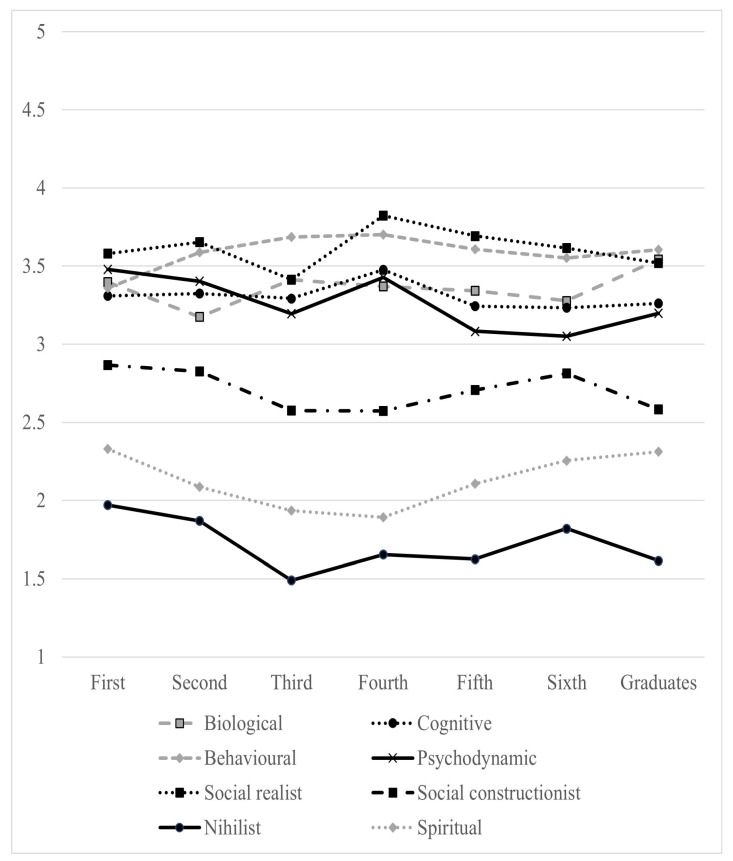
Mean aggregate endorsement scores by model and year of education. 1 = strongly disagree, 3 = neutral, 5 = strongly agree (see text).

The analysis by academic year is summarized in Table 4 and Figure 2. It also showed that there are significant differences in the endorsement of the psychodynamic model (*p* for trend =0.001) and the nihilist model (*p* for trend =0.046). Compared with students from the sixth year of their degree, first-year students reported higher scores for the psychodynamic model (LAD −0.43, *p* = 0.046). The endorsement of the nihilist model was higher in the first academic year than in the third year (LAD −0.48, *p* = 0.017).

## 4. Discussion

The aim of this study was to explore the conceptualization of depression among medical students and to determine whether there are differences between different academic years. Our findings show that medical students have a complex conceptualization of depression, combining several premises from different models, irrespective of their academic year. The only differences found across academic levels were for the psychodynamic model (less endorsement in more advanced years) and nihilist model (less endorsement in the third year than in the first year).

One of the main findings of our study was the variety of concepts used by the students to understand depression. According to the most endorsed items concerning its etiology, classification, treatment and research, medical students conceptualize depression as a consequence of social circumstances or conditions (social realist model) and they report that it is best approached by the study of abnormal behavior (behavioral model), its research should involve the discovery of biological markers and the effects of biological interventions (biological model) and it should be treated by challenging and restructuring maladaptive thoughts and beliefs (cognitive model). These four models are, together with the psychodynamic model, the most endorsed perspectives throughout the medical degree. This social awareness of the students, combined with other psychological and biological perspectives, suggest that the biopsychosocial model is the most dominant approach taken among medical students.

Despite the scarcity of studies and their methodological heterogeneity, our findings are in line with previous research. In a study conducted in 1998 [20], students considered both biological and psychological causes of depression important and found both psychiatric drugs and psychotherapy reliable. Similar results were found in another study carried out in 2005 [21]. A recently published study conducted with different mental health professionals and students also found a complex and multidimensional conceptualization of mental illnesses [15]. Finally, studies using the MAQ among psychiatrists [16], psychologists [17] and laypeople [18] also support a multidimensional approach to depression.

The biopsychosocial model appears as the most extensive approach in psychiatry [22]. We have deliberately referred to this perspective as an “approach” and not as a paradigm because it represents a form of eclecticism which combines the premises of different models. Engel proposed the biopsychosocial model in reaction to the rise of the biological model [9]. However, this proposal was not a new paradigm, as it did not develop a conceptual articulation that allowed for a coherent combination of these different perspectives [23]. Some of the criticism of this model includes the fact that the different formulations of the model tend to be vague and abstract, which prevents its true integration [22].

In daily practice, this lack of integration has masked a “bio-bio-bio” approach to mental health problems [3], therefore achieving the opposite goal of Engel’s proposal. This fact is related to Kuhn’s theory about the incommensurability of different scientific paradigms due to conceptual and methodologic differences [3,6] and is a powerful reason behind the importance of studying the underlying philosophies in psychiatry.

On the other hand, the least endorsed perspectives were the nihilist and spiritual models. The nihilist model represents a perspective which rejects the contribution of science to the understanding of depression, the competence of the mental health professionals and the utility of psychiatric or psychological treatments [16]. Therefore, the endorsement of any other paradigm, either biological, psychological or social (and besides the spiritual model) is inconsistent with its statements. Likewise, the spiritual model deviates from a scientific approach to the study of mental disorders, and its rejection can be read as another sign of the students’ scientific commitment.

Compared with the results reported by previous studies using the same instrument [16,17,18], our findings suggest that medical students show higher support for the biological model than laypeople and psychologists, but less than psychiatrists. This might be explained by the predominantly biological approach within medical education, placing students at the biological end of the biological–psychosocial continuum. One interesting result is that medical students abandon their proximity to the psychiatrists’ position in favor of the psychologists’ social models. In fact, their support of these models is higher than the support found in psychiatrists [16]. This would suggest a moment during medical education in which social awareness is lost in favor (or without change) of a more biological position. In our study, we did not find such a shift during students’ medical degree, thus it is possible that this shift might take place during residency.

It is important to note that the differences found with the cited previous studies might also be due to different characteristics (age, education, profession, etc.), cultures, settings and time. Thus, these conclusions should be treated cautiously.

One of our main findings was the almost complete absence of differences in the support of different models across academic years. This contrasts with two previous studies carried out in 1985 [24] and 1993 [25], which reported a shift to a more biological position after a six-week psychiatry course. Similarly, a recent study [21] found a change in the beliefs of students after a six-week psychiatry rotation.

We found significant changes in only two of the eight models: the psychodynamic and nihilist models. In both cases, the variation was modest: the differences were less than 0.5 Likert scale points. In our study, first-year students more strongly endorsed the nihilist model than third-year students. At the time of the assessment, the students in their third year had passed their psychiatry examination, completing their education in mental health (after studying medical psychology in their second year). Their recent exposure to this content could justify the increase in their trust towards the discipline, even from its already high level, as mentioned above. A previous study also found an increase in confidence in psychological and psychiatric treatments after a psychiatry clerkship [21].

The psychodynamic model shows a more pronounced evolution that goes beyond the variations between academic years and represents a more consistent tendency. This model is the only one that shows a significant difference by level of education. It receives its maximum support in the first year, when students have not yet studied any mental health subjects. This support decreases after studying psychiatry, a decrease which is maintained until the last academic year. The evolution of the endorsement of this model in medical students is identical to its historical evolution. After being the main paradigm during the 20th century, it was displaced by the biomedical model [3]. Similarly, the students’ support of this model decreases as their contact increases with current psychiatric theory and practice, which is mainly biomedical.

Finally, the evolution of the variables added to explore the accessibility of the questionnaire is remarkable. The second level of education (post-psychiatry level), and especially the third year of the degree, represents a point of inflexion in the psychiatric culture of students. In that process, their understanding of the biological, cognitive and behavioral models, as well as their comprehension of the disorder, seems to become more robust. Those are the prevailing models in current psychiatry and, therefore, the main models taught in their degree. It seems that the study of psychiatry increases their comprehension of its most important paradigms and, at the same time, encourages their confidence in the discipline.

Some concerns about the sensitivity of the questionnaire in detecting differences between students deserve a more detailed comment. The perspectives used to understand different concepts in psychiatry are not always coherently embedded. In fact, it is common to hold complex views and even make inconsistent statements [15]. Therefore, as the MAQ obtains a mean aggregate endorsement score that combines their support of only four aspects of each model, a significant change in one of the items could not be properly captured when merged with the other three items. This could help explain the absence of more or greater differences.

The original questionnaire evaluates the conceptualization of other mental disorders, that is, schizophrenia, generalized anxiety disorder and antisocial personality disorder. The evidence shows that mental disorders are not understood as homogenous entities [7,16,18]. Thus, future studies using the full questionnaire on medical students might also be of interest. Finally, the relatively homogenous results found across different years of education indicate a mild or null influence of the study of psychiatry on the conceptualization of depression among medical students. Qualitative studies might help further explore the reasons behind these results.

The preliminary nature of this work entails some limitations. First, due to its observational design, our work points to correlations and may be a source of future hypotheses, but it does not allow us to causally explain the differences between educational levels.

Second, the psychometric properties of the original English version of the MAQ are not fully known. Also, despite the fact that our translated survey obtained good results in its preliminary validation with experts and students, future studies are needed to further investigate the psychometric properties and construct validity of both the English and Spanish versions of the questionnaire. In this sense, in addition to classical methods (e.g., Cronbach’s Alpha), new tools that have been applied, with interesting results, to symptomatic scales of depression (e.g., Network Analysis [26], Item Response Theory [27]) could contribute to the validation of the scales of its conceptualization.

Second, the comparison between different professional groups (psychiatrists, psychologists, laypeople and medical students) was originally based on studies developed in the UK, limiting its validity. To place our results in a valid context, it would be necessary to study the conceptualization of mental disorders in Spanish laypeople, doctors from different specialties (i.e., psychiatrists and GPs) and psychologists. Interesting conclusions could arise from this analysis, especially about the influence of different stages of medical education, differences between medical specialties and professions and the divergences between health professionals, patients and laypeople.

Finally, some concerns might prevent us from generalizing these results to the medical student population. First, our response rate was low, although in line with other similar studies. For example, a 2020 study of medical students’ attitudes towards people with mental illness and psychiatry conducted through an online survey in three European universities showed a response rate between 6.5 and 19.4% [28]. Other possible sources of bias include a somewhat more feminized sample and the fact that the study was carried out at a single institution.

Despite these limitations, and to the best of the authors’ knowledge, this is the first published research studying medical students’ endorsement of eight different models of psychiatry. It is also the first work of this nature carried out in Spain. We have explored the differential attitudes of a range of students, from first-year students to recently graduated doctors, a topic scarcely studied before. Additionally, we developed the Spanish version of the MAQ, which has preliminary good psychometric properties. This questionnaire is the only survey that explores different the psychiatry models that have been previously applied to different populations. Our study provides further evidence on the subject by analyzing model conceptualization among Spanish medical students.

## 5. Conclusions

The integration of the wide range of phenomena included in the study of human behavior is a theoretical, conceptual and philosophical challenge. The way human suffering is conceptualized has a direct impact on the possibility of its study, explanation and alleviation [4]. Thus, understanding our knowledge of these aspects appears to be a moral responsibility [4].

Given the social and health importance of mental disorders and their frequent observation in the clinical practice of any medical specialty, the study of how medical students conceptualize mental disorders during their university degree emerges as a priority, as other authors have previously suggested [4,14,29].

With this work, we have begun to investigate this scarcely studied area. Through the translation and preliminary validation of the Maudsley Attitudes Questionnaire, we have found that medical students understand depression from a multidimensional and complex perspective that includes concepts belonging to different psychological, biological and social models. Apparently, the influence of medical education on this conceptualization is reduced: first-year students and recently graduated doctors seem to understand depression in a very similar way. The education received increases their confidence in psychiatry, reduces their endorsement of the psychodynamic model and improves their understanding of the biological, cognitive and behavioral models of depression, the most important models of the discipline.

Future research on the psychometric properties of the MAQ and its application in different populations could significantly enrich our knowledge on this important topic. 

This would allow us to detect the educational needs of students in the conceptual competence of psychopatology and to design the best strategy to address them.

## Figures and Tables

**Table 1:** Original questionnaire items by model (number of the item indicates the order of the item’s appearance in the questionnaire).

Biological	
1.	The disorder results from brain dysfunction
6.	The ideal classification of the disorder would be a pathophysiological one
9.	The appropriate study of the disorder involves discovery of biological markers and the effects of biological interventions
17.	Treatment of the disorder should be directed at underlying biological abnormalities
**Cognitive**	
15.	Maladaptive thoughts and beliefs are normally distributed in the population and it is the extreme ends of this distribution that account for the disorder
24.	The disorder is nothing other than the sum of maladaptive thoughts, beliefs and behaviors
20.	The study of the disorder should concentrate on understanding cognitive distortions and reasoning errors
7.	The disorder should be treated by challenging and restructuring maladaptive thoughts and beliefs
**Behavioral**	
31.	The disorder results from maladapted associative learning
3.	The disorder is best approached through the study of abnormal behavior
11.	Studying the associations between antecedents and consequents in patients’ behavior is the best basis for modification of the disorder
19.	The behavioral problems in the disorder are best modified by associating new responses to a given stimulus
**Psychodynamic**	
26.	The disorder results from the failure to successfully complete developmental psychic stages
18.	The disorder is due to unconscious factors (as defined psychodynamically)
22.	The structure of the disordered psyche and its unconscious mechanisms is best understood by a study of individual cases
28.	Treatment of the disorder requires resolution of disturbed early object relationships
**Social realist**	
14.	Social factors such as prejudice, poor housing and unemployment are the main causes of the disorder
2.	The disorder arises as a consequence of social circumstances or conditions
5.	The research into the disorder should focus on the identification of causative social factors
29.	Government policies to reduce prejudice, poor housing and unemployment are the way to eradicate the disorder
**Social constructionist**	
16.	There is no universal classification of disorder, only culturally relative classifications
32.	The disorder is a culturally determined construction that reflects the interests and ideology of socially dominant groups
13.	The disorder can only be understood in the context of local meanings and these meanings cannot be extrapolated to universal classifications
10.	Treatment of the disorder should be based on whatever folk treatments and models are accepted as appropriate by the patient and their local community
**Nihilist**	
23.	Attempts to scientifically explain the disorder have resulted in no significant knowledge
27.	All classifications and ‘ treatments ’ of the disorder are myths
12.	Mental health professionals have no ‘ expertise ’ of the disorder over and above anyone else
4.	The management of the disorder is best left to the resources of the individual
**Spiritual**	
8.	Neglecting the spiritual or moral dimension of life leads to the disorder
30.	The disorder is better understood through religious or spiritual insights
25.	Consulting a spiritual authority can give a better understanding of the disorder than psychiatry
21.	Adherence to religious or spiritual practice is the most effective way of treating the disorder

**Table 2:** Description of the sample. SD: standard deviation. The shaded rows, which group the rows immediately above them, are the student clusters explained in Section 2.2.3.

Year or Level	Participants n (%)	Male n (%)	Female n (%)	Age Mean (SD)
First year	34 (15.7%)	3 (8.8%)	31 (91.2%)	18.8 (0.6)
Second year	23 (10.7%)	3 (13%)	20 (87%)	20 (0.7)
Pre-psychiatry	57 (26.4%)	6 (10.5%)	51 (89.5%)	19.2 (0.9)
Third year	23 (10.7%)	8 (34.8%)	15 (65.2%)	20.7 (0.6)
Fourth year	21 (9.7%)	5 (23.8%)	16 (76.2%)	22.5 (1.7)
Fifth year	30 (13.9%)	5 (16.7%)	25 (83.3%)	23.2 (1.8)
Post-psychiatry	74 (34.3%)	18 (24.3%)	56 (75.7%)	22.2 (1.8)
Sixth year	61 (28.2%)	16 (26.2%)	45 (73.8%)	24.7 (3.5)
Graduates	24 (11.1%)	5 (20.8%)	19 (79.2%)	24.9 (1)
Pre-residency	85 (39.4%)	21 (24.7%)	64 (75.3%)	24.8 (3)

**Table 3:** Mean aggregate endorsement scores by model and level of education. SD: standard deviation. Statistically significant results are marked with an asterisk.

Model	Pre-Psychiatry Mean (SD)	Post-Psychiatry Mean (SD)	Pre-Residency Mean (SD)	*p*
Biological	13.23 (2.46)	13.49 (2.73)	13.41 (2.30)	0.667
Cognitive	13.26 (1.96)	13.30 (1.91)	12.96 (2.10)	0.383
Behavioral	13.81 (1.97)	14.64 (2.03)	14.25 (1.05)	0.15
Psychodynamic	13.79 (2.20)	12.86 (2.06) *	12.36 (2.06) *	<0.001
Social realist	14.44 (2.26)	14.57 (2.27)	14.35 (1.94)	0.816
Social constructionist	11.40 (2.36)	10.51 (2.31)	10.99 (2.40)	0.305
Nihilist	7.72 (2.44)	6.36 (1.79)	7.05 (2.12)	0.063
Spiritual	8.93 (2.26)	7.97 (2.15)	9.08 (2.44)	0.655

**Table 4:** Mean aggregate endorsement scores by model and year of education. SD: standard deviation. Statistically significant results are marked with an asterisk.

	First Mean (SD)	Second Mean (SD)	Third Mean (SD)	Fourth Mean (SD)	Fifth Mean (SD)	Sixth Mean (SD)	Graduates Mean (SD)	*p*
Biological	13.59 (2.19)	12.70 (2.77)	13.65 (3.02)	13.48 (2.80)	13.37 (2.51)	13.11 (2.37)	14.17 (1.95)	0.348
Cognitive	13.24 (1.71)	13.30 (2.32)	13.17 (1.90)	13.90 (1.79)	12.97 (1.96)	12.93 (2.09)	13.04 (2.14)	0.435
Behavioral	13.44 (1.78)	14.35 (2.15)	14.74 (2.40)	14.81 (1.50)	14.43 (2.10)	14.21 (1.71)	14.42 (1.50)	0.200
Psychodynamic	13.91 (2.18)	13.61 (2.27)	12.78 (1.93)	13.71 (1.62)	12.33 (2.28)	12.20 (2.10) *	12.79 (1.93)	0.001
Social realist	14.32 (2.45)	14.61 (1.99)	13.65 (2.04)	15.29 (2.37)	14.77 (2.21)	14.46 (1.95)	14.08 (1.93)	0.964
Social constructionist	11.47 (2.15)	11.30 (2.69)	10.30 (2.78)	10.29 (2.33)	10.83 (1.90)	11.25 (2.40)	10.33 (2.32)	0.195
Nihilist	7.88 (2.56)	7.48 (2.29)	5.96 (1.52) *	6.62 (2.11)	6.5 (1.74)	7.28 (2.27)	6.46 (1.53)	0.046
Spiritual	9.32 (2.43)	8.35 (1.87)	7.74 (2.28)	7.57 (1.96)	8.43 (2.16)	9.02 (2.32)	9.25 (2.77)	0.420

## Data Availability

The raw data supporting the conclusions of this article will be made available by the authors on request.
